# Study protocol: a dose-escalating, phase-2 study of oral lisdexamfetamine in adults with methamphetamine dependence

**DOI:** 10.1186/s12888-016-1141-x

**Published:** 2016-12-01

**Authors:** Nadine Ezard, Adrian Dunlop, Brendan Clifford, Raimondo Bruno, Andrew Carr, Alexandra Bissaker, Nicholas Lintzeris

**Affiliations:** 1 Alcohol and Drug Service, O’Brien Centre, St Vincent’s Hospital, Sydney, Darlinghurst, 2010 NSW Australia; 2 St Vincent’s Hospital Clinical School, Faculty of Medicine, UNSW, Sydney, Australia; 3 Drug and Alcohol Clinical Services, Hunter New England Local Health District, Newcastle Community Health Centre, Newcastle, 2302 NSW Australia; 4 School of Medicine, University of Tasmania, Private Bag 30, Hobart, 7001 TAS Australia; 5 Centre for Applied Medical Research, St Vincent’s Hospital, 390 Victoria Street, Darlinghurst, 2010 NSW Australia; 6 South East Sydney Local Health District, The Langton Centre, 591 South Dowling St, Surry Hills, 2010 NSW Australia; 7 Discipline of Addiction Medicine and Lambert initiative in Cannabinoid Therapeutics, University of Sydney, Sydney, 2006 NSW Australia

**Keywords:** Methamphetamine, Study protocol, Lisdexamfetamine, Pharmacotherapy, Dose-finding, Stimulant use disorder

## Abstract

**Background:**

The treatment of methamphetamine dependence is a continuing global health problem. Agonist type pharmacotherapies have been used successfully to treat opioid and nicotine dependence and are being studied for the treatment of methamphetamine dependence. One potential candidate is lisdexamfetamine, a pro-drug for dexamphetamine, which has a longer lasting therapeutic action with a lowered abuse potential. The purpose of this study is to determine the safety of lisdexamfetamine in this population at doses higher than those currently approved for attention deficit hyperactivity disorder or binge eating disorder.

**Methods/design:**

This is a phase 2 dose escalation study of lisdexamfetamine for the treatment of methamphetamine dependence. Twenty individuals seeking treatment for methamphetamine dependence will be recruited at two Australian drug and alcohol services. All participants will undergo a single-blinded ascending-descending dose regime of 100 to 250 mg lisdexamfetamine, dispensed daily on site, over an 8-week period. Participants will be offered counselling as standard care. For the primary objectives the outcome variables will be adverse events monitoring, drug tolerability and regimen completion. Secondary outcomes will be changes in methamphetamine use, craving, withdrawal, severity of dependence, risk behaviour and other substance use. Medication acceptability, potential for non-prescription use, adherence and changes in neurocognition will also be measured.

**Discussion:**

Determining the safety of lisdexamfetamine will enable further research to develop pharmacotherapies for the treatment of methamphetamine dependence.

**Trial registration:**

Australian and New Zealand Clinical Trials Registry ACTRN12615000391572 Registered 28^th^ April 2015.

## Background

### Background

Amphetamine type stimulants, including methamphetamine, present a global public health concern. The second most commonly used illicit drug worldwide, approximately 34 million people aged 15–64 (range 14–56 million) were estimated to be using amphetamine type stimulants in 2010 [1] with 17 million people (range 14–21 million) estimated to have dependence [2]. Problems from stimulant use in Australia are largely related to methamphetamine, and include psychosis (often requiring hospital admission), dependence, injecting-related risks, high-risk sexual practices, psychological disturbances, and acute cardiovascular and cerebrovascular events [3]. The current standard care for methamphetamine dependence comprises psychosocial interventions, which shows modest rates of induction and retention with many effects lost at follow-up [4, 5].

Agonist-type pharmacotherapies are candidates to improve treatment outcomes for methamphetamine dependence. By mimicking the pharmacodynamic effects of methamphetamine [6], agonists may ameliorate withdrawal and cravings, attenuate the positive effects of methamphetamine use, enable use reduction or abstinence and/or increase engagement with treatment [7]. Additional harm reduction may be achieved by replacing illegal drug use with a legal, orally administered and regulated alternative. Agonist-type pharmacotherapies have successfully promoted use reduction and treatment retention in opioid dependence [8] and smoking abstinence for nicotine dependence [9]. Emerging evidence suggests that dexamphetamine may be effective in reducing cocaine use among heroin-maintained individuals [10] and a statistical trend in improving sustained cocaine abstinence has been observed in trials of dexamphetamine, modafanil and bupropion [11, 12].

Both immediate and extended release forms of dexamphetamine have  been trialled as agonist-type pharmacotherapies for methamphetamine dependence [13–16]. The active component dexamphetamine induces neurotransmitter release with a similar pattern to methamphetamine. Randomized controlled trials using 60 to 110 mg of dexamphetamine have shown a statistically significant decrease in amphetamine withdrawal (g ~ 0.57 to 0.62 [13, 14]) and craving (g ~ 0.59 [14]) and an increase in treatment retention (g ~ 0.72 [13, 14]). Although there was a significant decrease in methamphetamine use from baseline, the trials appear insufficiently powered to elicit a difference from placebo.

Methamphetamine dependence research is currently limited by a low number of published, randomized controlled trials with adequate sample size, duration and follow-up [17]. More targeted therapy should also be considered, with increased stimulant dependence at baseline, longer dosing intervals, higher doses and longer duration of treatment correlating with better clinical outcomes for psychostimulant dependence [7].

Lisdexamfetamine dimesylate (LDX), a dexamphetamine pro-drug, has been developed for the treatment of attention deficit hyperactivity disorder and binge eating disorder [18]. Once ingested, LDX undergoes rate-limited hydrolysis by enzymes within red blood cells to release l-lysine and dexamphetamine [19]. The in vivo and rate limited conversion of LDX provides longer lasting therapeutic action with pre-clinical studies showing a slower onset and longer lasting dopamine release in rat striatum compared to similar agonist formulations, immediate release dexamphetamine and methylphenidate [20, 21]. Pharmacokinetic studies have shown longer time to peak, lower maximal concentration and longer duration of action compared to immediate release dexamphetamine [22]. The pro-drug also benefits from a reduced diversion or abuse liability with the kinetics of dexamphetamine remaining consistent between intranasal, intravenous or oral administration [23, 24]. In clinical studies LDX has displayed a lower subjective drug liking by stimulant users when compared to immediate release dexamphetamine and methylphenidate [24, 25].

### Rationale for study

LDX has the potential to improve treatment outcomes for methamphetamine dependence, however there have been no published trials addressing this question. Doses of 100 to 250 mg LDX may be required to match the 60 to110mg used in trials of dexamphetamine for methamphetamine dependence. These doses are higher than the 70 mg LDX currently approved for attention deficit hyperactivity disorder and binge eating disorder. In order to inform the design of phase 3 trials on the efficacy of LDX as a treatment for methamphetamine dependence, the safety of LDX in this population at the required therapeutic doses needs to be established.

The four most common side effects noted by participants in clinical trials of LDX are decreased appetite (27%), insomnia (27%), dry mouth (26%) diarrhea/nausea (both 7%) in adults with ADHD [18] and dry mouth (36%), headache (14%), insomnia (14%) and decreased appetite (12%) in adults with binge eating disorder [26]. LDX is contraindicated in persons with known allergy and with concurrent or use within the previous 14 days of monoamine oxidase inhibitors (MAOIs) [18]. There are also warnings and precautions for serious cardiovascular reactions, blood pressure and heart increases, psychiatric adverse reactions and peripheral vasculopathy (including Raynaud’s phenomenon). On this basis, known contraindications, pre-existing cardiovascular disease and peripheral vasculopathy will be exclusion criteria for the trial, and measurement tools specific to blood pressure, heart rate, psychiatric symptoms, weight and insomnia will be administered (see Table 1). Other adverse effects will be elicited by non-directive questioning at study visits.

**Table 1:** Schedule of enrolment, intervention and assessments based on SPIRIT 2013 guidelines [27]

Week	−2	−1	1	2	3	4	5	6	7	8	12
Informed Consent	●										
Eligibility	●										
Demographic data	●										
Height	●										
Medical & Psychiatric History	●										
Self-reported drug use	●										
Concomitant medications	●										
Intervention											
Treatment as usual (counselling) offered weekly											
Dose of *Lisdexamfetamine* (mg)-dispensed daily		100	150	200	250	250	200	150	100	End	FU
Baseline Measures											
MOCA, AUDIT, WTAR, Wender Utah Scale		●									
Primary Outcomes											
Brief Psychiatric Scale: psychosis & hostility items		●	●	●	●	●	●	●	●	●	●
Vital Signs^a^ (Blood pressure, pulse, temperature)	●	●	●^t^	●^t^	●^t^	●^t^	●	●	●	●	●
Insomnia Severity Index		●		●		●		●		●	●
Patient Health Questionnaire 15		●				●				●	●
Patient Health Questionnaire 9		●		●		●		●		●	●
Generalized Anxiety Disorder 7		●		●		●		●		●	●
Weight (in kilograms)	●	●				●				●	●
Adverse Events Log		●	●	●	●	●	●	●	●	●	●
Electrocardiogram	●			●		●					
TSQM-side-effects item			●	●	●	●	●	●	●	●	●
Proportion completing dose escalation phase						●					
Secondary Outcomes											
Substance Use TLFB-MA (Days Used)	●	●	●	●	●	●	●	●	●	●	●
Urine Drug Screen (positive MA)	●^d^	●	●	●	●	●	●	●	●	●	●
Substance Use TLFB-MA (Days Used)		●	●	●	●	●	●	●	●	●	●
Urine Drug Screen^c^	●^d^	●	●	●	●	●	●	●	●	●	●
TSQM-effectiveness item		●	●	●	●	●	●	●	●	●	●
Visual Analogue Scale for MA craving		●^t^	●^t^	●^t^	●^t^	●^t^	●	●	●	●	●
Amphetamine Withdrawal Questionnaire		●^t^	●^t^	●^t^	●^t^	●^t^	●	●	●	●	●
Severity of Dependence Scale		●								●	●
Adapted Opiate Treatment Index (HIV & crime scales)		●				●					●
TSQM-convenience & global satisfaction items		●	●	●	●	●	●	●	●	●	●
Proportion discontinuing once enrolled			●	●	●	●	●	●	●	●	
Price would Pay		●^b^	●^b^	●^b^	●^b^	●^b^					
Similarity to MA Visual Analogue Scale		●^b^	●^b^	●^b^	●^b^	●^b^					
Drug Effects Questionnaire 5		●^b^	●^b^	●^b^	●^b^	●^b^					
Acute Subjective Response to Substances		●^b^	●^b^	●^b^	●^b^	●^b^					
Trail-making Test		●^t^	●^t^	●^t^	●^t^	●^t^					●
Rey Auditory Verbal Learning Task		●^t^	●^t^	●^t^	●^t^	●^t^					●
Digit-span sequencing		●^t^	●^t^	●^t^	●^t^	●^t^					●
Flankers Test with no-go		●		●		●					●
Digit Symbol Substitution		●		●		●					●
Rapid Information Processing		●		●		●					●

*Abbreviations*: ^*t*^timed trough/peak measurements prior to study drug administration/4 h post, *TSQM* Treatment Satisfaction Questionnaire for Medications, *MOCA* Montreal Cognitive Assessment, *AUDIT* Alcohol Use Disorder Identification Test, *WTAR* Wechsler Test of Adult Reading, *MA* methamphetamine, *TLFB* Time-Line Follow Back questionnaire

^a^vital signs measured daily

^b^Measurement taken 4 h post study drug administration

^c^Urine drug screen for benzodiazepines, cannabis, cocaine, methadone, morphine/heroin and oxycodone

^d^Two urines positive for methamphetamine required for eligibility

## Methods

### Design

**Fig. 1:**
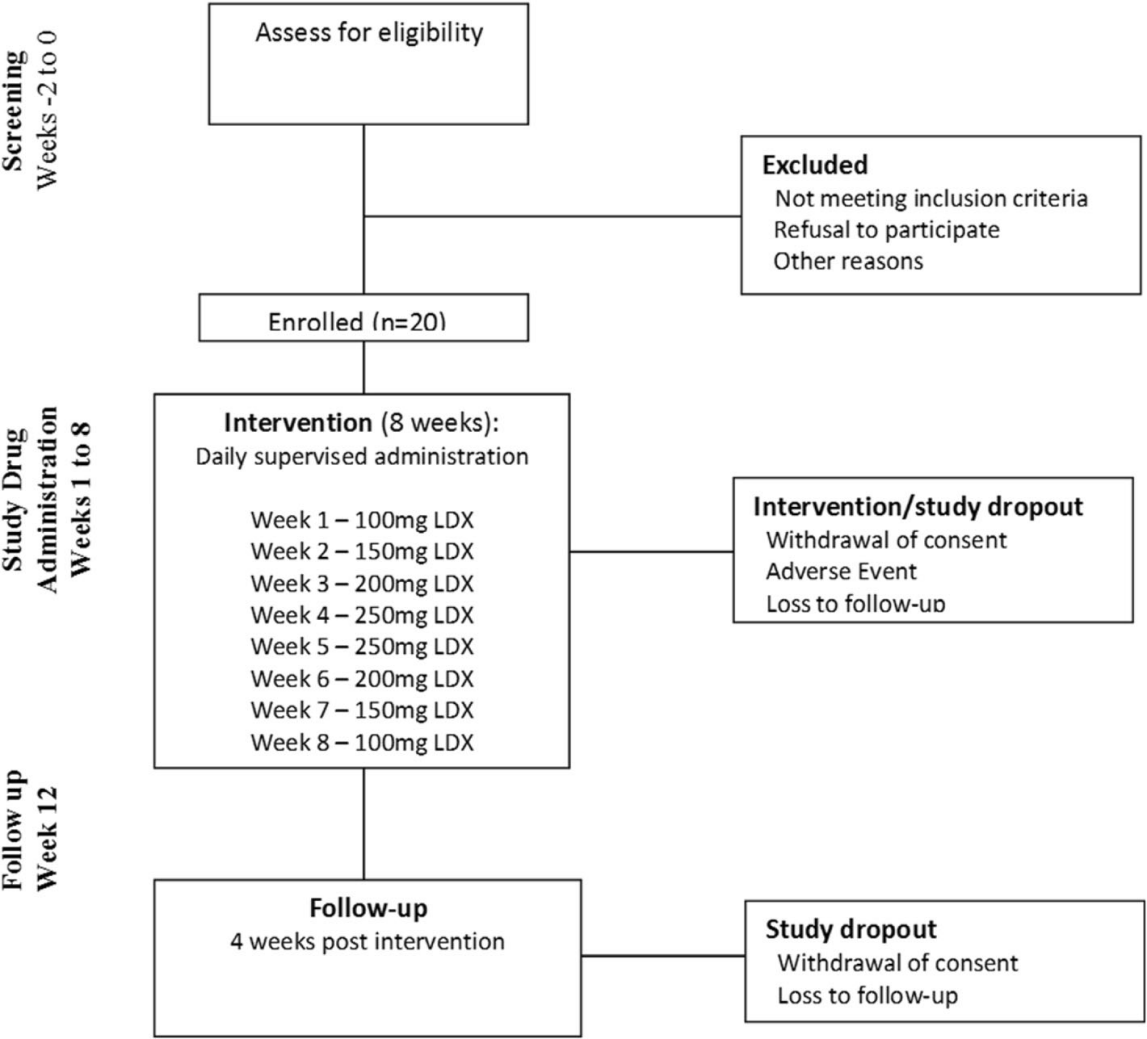
Study flow diagram

The study is a phase 2, single group, outpatient study with a study period of 14 weeks, inclusive of screening and follow up. Screening will occur over 2 weeks (weeks −2 and −1), and eligible participants commenced on the escalation/de-escalation phase (week 1). Escalation of LDX will occur over 4 weeks, beginning at 100 mg/day, with weekly increases of 50 mg to a maximum 250 mg/day. After 2 weeks at 250 mg/day LDX, the dose of LDX will be reduced weekly by 50 mg/day, ceasing after 1 week at 100 mg/day. A follow up study visit will occur in week 12, 4 weeks after cessation of study drug. The study aims to enrol a sample of twenty participants who are seeking treatment for methamphetamine dependence. A diagrammatic overview of the study is given in Fig. 1. The protocol has been designed in accordance with CONSORT SPIRIT guidelines [27].

### Study objectives

The study’s primary objectives are to describe the safety, tolerability and regimen completion of ascending doses of LDX in adults with methamphetamine dependence.

Secondary outcomes include changes in methamphetamine use, craving, withdrawal, severity of dependence, risk behaviour and in other substance use. Medication acceptability, potential for non-prescription use, adherence and changes in neurocognition will also be measured.

### Inclusion criteria

Participants in the trial are aged at least 18 years or over who fulfil ICD10 criteria [28] for methamphetamine dependence and have ≥2 years of problematic methamphetamine use (self-reported), for which they are currently seeking treatment. Each participant must have a self-reported methamphetamine use of ≥14 days in the 28 days prior to consenting. A urine specimen is obtained weekly for 2 weeks prior to the commencement of study drug, and screened to verify methamphetamine use. Participants must be willing and able to comply with the requirements of the study and be able to provide written, informed consent.

### Exclusion criteria

Exclusion criteria are: use of dexamphetamine in the previous 4 weeks; sensitivity or previous adverse reaction to LDX; or current use of medications that could interact with LDX (venlafaxine, desvenlafaxine, monoamine oxidase inhibitors (MAOI); MAOI use in the previous 14 days, a history of moderate to severe hypertension (well-controlled mild to moderate hypertension on a single antihypertensive agent permitted), severe and symptomatic peripheral vascular disease or Raynaud’s phenomenon; significant prior or symptomatic cardiovascular disease; glaucoma; hyperthyroidism; phaeochromocytoma; motor and phonic tics; Tourette’s syndrome; unstable alcohol or other substance use as assessed by the principal investigator; high suicide risk; voicing suicidal ideation; active psychosis (past history of psychosis permitted on review of a psychiatrist); severe agitation and severe cognitive impairment. Women who are lactating, pregnant or of childbearing potential who are not willing to avoid becoming pregnant during the study will also be excluded.

### Identification of participants and informed consent

Initial recruitment is made through clients registered with stimulant treatment programs at each site. Ethics Committee-approved print media containing poster and flyer displays are displayed at each site targeting the sample population including other drug and alcohol services, community/NGOs and local general practices surgeries. If necessary, web-based advertising may be used, including promotion via the stimulant treatment program’s established social media pages. Screening visits are arranged upon approach by interested individuals who satisfy pre-screening assessment of basic inclusion and exclusion criteria.

At the screening visit, the principal investigators at each site (specialists in addiction medicine) assess the eligibility of potential participants. The trial is explained verbally and in written form including the risks and burdens of participating before written consent is obtained. Screening involves a medical and substance use history as well as a physical and mental state examination, electrocardiogram (ECG) and urinary human chorionic gonadotropin (hCG) where applicable to verify that the individual meets the eligibility criteria. Two urine drug screens, at least a week apart, are used to confirm methamphetamine use.

### Intervention

The LDX dosing period is up to 8 weeks and consists of daily, supervised outpatient oral dose administration at the participants’ respective site. Dose escalation and de-escalation range from 100 mg per day to a maximum 250 mg per day, with changes weekly to allow for achievement of steady state [29]. Both the participants and drug administration personnel are blinded to date, amount and direction of dose change. To achieve this, the dose comprises 5 identical size 0 capsules each containing either 50 mg LDX or placebo according to the dose escalation phase.

Additionally, participants are offered weekly counselling with trained and supervised counsellors at the Stimulant Treatment Programs as current standard of care for methamphetamine dependence, though attendance is not mandatory.

### Safety and discontinuation

Specific adverse events resulting in participant discontinuation are: pregnancy checked through monthly urinary hCG tests where applicable; clinically significant hypertension (two consecutive readings of systolic blood pressure >160 mmHg or diastolic blood pressure >100 mmHg within an hour); persistent tachycardia (>120 beats per minute for >60 min) or clinically significant psychotic symptoms (as assessed by the principal investigator). Additional unscheduled visits occur if there is any concern regarding possible toxicity. Further reasons for participant withdrawal are the observed diversion of study drug, withdrawal of consent, missing three or more doses in a 7 day period or missing more than one extended data collection visit. An independent data safety and monitoring board has been established to ensure adequate safety outcomes and management of the study drug for the continuation of the study. It will first meet after 5 participants have commenced the study drug and completed up to the end of the dose escalation period (or discontinued prior) and then twice yearly. Any adverse effects experienced by participants will be treated by the study team, or referred to a specialist as required.

### Assessment schedule

Baseline assessments and demographic data are established in the screening visit or before LDX dispensing on the first day of the LDX intervention. Baseline substance use history is recorded, and the Alcohol Use Disorder Identification Tool (AUDIT) used to establish alcohol use patterns [30]. During the 8-week dosing period, daily assessment of vital signs and the recording of treatment emergent adverse events will be undertaken at drug dispensing. In weeks 1–4, one extended visit per week occurs for detailed data collection concerning the safety and efficacy outcomes. Selected timed assessments (as outlined in Table 1) are conducted pre-dose dispensing (trough level, equivalent to LDX steady state) and four hours post dosing for peak levels [31]. In the follow up period 4 weeks after the final dose, an additional extended visit will assess the same safety and efficacy outcomes measured in the dose-escalation period. The participant is reimbursed for extended visits with the equivalent of $80 in a supermarket voucher. Assessment raters will be trained and supervised by the study psychologist (RB), an experienced professor of psychology, and tests will be administered using standardised scripts.

### Data collection

#### Primary outcomes

Treatment emergent adverse events (TEAEs) and vital signs (blood pressure, pulse rate, respiratory rate and temperature) are recorded daily for the 8-week dosing period. In weeks 1–4, at each data collection visit, vital signs are recorded at trough and peak times. An ECG is performed at weeks 2 and 4, and weight in kilograms measured at week 4 and at follow-up. Differences in symptoms of psychosis and hostility are obtained using the psychosis and hostility items of the Brief Psychiatric Rating Scale [32], changes in sleep quality measured using the Insomnia Severity Index [33] and changes in somatic symptoms, depression and anxiety measured with the Patient Health Questionnaire 15 [34], Patient Health Questionnaire 9 [35] and the Generalized Anxiety Disorder 7 [36] questionnaire respectively.

Tolerability of LDX is measured by the Treatment Satisfaction Questionnaire for Medications (TSQM) side effects item [37]. Completion rates are calculated by the proportion of participants completing escalation to steady-state of 250 mg at week 4.

#### Secondary objectives

Change in methamphetamine use is recorded with the substance use Timeline Follow-back (TLFB) interview [38] and urine drug testing. Urine screening for amphetamine-type substances is initially determined by immunoassay (cut-off of 300 ng/ml), before confirmation of drug type (methamphetamine, amphetamine or other) using gas chromatography-mass spectroscopy (cut-off of 150 ng/ml) [39]. Change in use of other substances of concern is also assessed by the TLFB interview, with urine screening to Australian Standard ASNZS4308 [39] for benzodiazepines, cannabis, cocaine, methadone, morphine/heroin and oxycodone. Change in risk behaviour is assessed through the Opiate Treatment Index Injecting and Crime risk questionnaires [40]. Craving and withdrawal symptoms are assessed with a visual analogue scale for craving [41] and the Amphetamine Withdrawal Questionnaire [42] at all data collection visits, including peak and trough times during the extended visits. Severity of dependence to methamphetamine is assessed with the Severity of Dependence Scale [43]. Participant rating of dose adequacy is assessed using the Treatment Satisfaction Questionnaire for Medication (TSQM) effectiveness item [37]. Acceptability of the study drug is assessed using TSQM convenience and global satisfaction items [37]. Potential for non-prescription use (sometimes referred to as abuse liability) is assessed using the Drug Effects Questionnaire [44], the Acute Subjective Response to Substances questionnaire, asking participants what price they would pay for the drug on the street and using a visual analogue scale for participants to rate study drug similarity to methamphetamine [45]. The frequency of the instruments used to assess secondary objectives is outlined in Table 1.

Neuropsychological measures taken at baseline are the Montreal Cognitive Assessment (MoCA) [46] and the Wechsler Test of Adult Reading (WTAR) [47]. The Wender Utah scale is used to screen for the presence of co-existing Attention-Deficit Hyperactivity Disorder [48]. Two separate batteries of neurocognitive testing, Set A and Set B, are administered according to the schedule set out in Table 1, which also outlines the neurocognitive domains measured by each instrument. Neurocognitive Set A is paper based, and consists of the Rey Auditory Verbal Learning Task [49], Trail-making test [50, 51], and Digit-Span Sequencing [52]. Neurocognitive Set B is administered using an electronic tablet device, and consists of Flankers task [53], Digit Symbol coding [54] and Rapid Visual Information Processing task [54].

#### Statistical analysis

Given the exploratory nature of the study, twenty participants will provide sufficient proof of the safety of the dose. While not powered to detect statistically significant effect sizes, findings will inform a planned randomised control trial of LDX for the treatment of methamphetamine dependence.

Descriptive statistics of proportion commenced on study drug who complete the study; proportion who achieve each dose; proportion who experience adverse event by type, severity and dose. Tolerability of LDX will be described using medians and interquartile ranges of TSQM side-effects scores. Continuous and categorical primary outcomes will be examined within a generalised linear mixed model framework [55]. Secondary outcomes measures changes in MA use, other substance use, craving, withdrawal, severity of dependence, risk behaviour scores and neurocognition will be tested for statistical significance using Wilcoxon rank-sum test for non-parametric data. The estimation of magnitude of effects of LDX on MA use will be calculated separately for those individuals that have and have not been adherent to counselling during the course of the study. Dose adequacy, medication acceptability, and potential for non-prescription use will be described using median scores and interquartile ranges at each dose. Analysis of urine drug results will follow established practice so that missing urine results are assigned as being positive for methamphetamine and the proportion of urines positive for methamphetamine will be reported. Missing data will be imputed using multiple imputation procedures.

## Discussion

Research related to pharmacotherapy options for the treatment of methamphetamine dependence has been often underpowered, with some concerns of under-dosing also noted [6]. The publication of study protocols such as this not only represents good clinical trial practice, but allows for the sharing of methodology in phase 2 studies in addiction pharmacotherapy research. Safety data for this population are essential to ensure dosing at optimal levels in larger scale trials. As abstinence from methamphetamine use for the period of the trial is not mandatory, the safety data elicited from this trial may be applied to more “real-world” scenarios, such as outpatient programs for stimulant users. Limitations of the study include the lack of a control group and the small sample size. While this is typical of numbers required for safety data, it limits the generalizability of the secondary efficacy measures. The trial is open-label, and non-randomised and thus participants may be subject to expectancy effects. To mitigate this, subjects and dispensing nurses are aware of the minimum and maximum doses, but blinded to the dose and to the escalation and de-escalation regimen. Though concurrent methamphetamine use is dependent on self-report, a validated tool (the TLFB interview) is used to obtain this data. Determining the safety and dose optimization of LDX within the target population will provide valuable data for phase 3 studies on the efficacy of LDX to treat methamphetamine dependence.

## Abbreviations

AUDIT: Alcohol use disorders identification test
CONSORT: Consolidated standards of reporting trials
ECG: Electrocardiogram
hCG: Human chorionic gonadotropin
LDX: Lisdexamfetamine
SPIRIT: Standard protocol items: recommendations for interventional trials
TEAE: Treatment emergent adverse events
TLFB: Time line follow back interview
TSQM: Treatment satisfaction questionnaire for medications
WTAR: Wechsler test of adult reading
